# Editorial: Cholesterol and Neurodegenerative Diseases - Pressing Questions and How to Address Them

**DOI:** 10.3389/fnagi.2022.948153

**Published:** 2022-06-08

**Authors:** Sandrine Betuing, Irina A. Pikuleva, Joseph M. Castellano

**Affiliations:** ^1^Neuroscience Paris Seine, Institut de Biologie Paris-Seine, Sorbonne Université, Faculté des Sciences et Ingénierie, Paris, France; ^2^Centre National de la Recherche Scientifique, UMR8246, Paris, France; ^3^Institut National de la Santé et de la Recherche Médicale, U1130, Paris, France; ^4^Department of Ophthalmology and Visual Sciences, Case Western Reserve University, Cleveland, OH, United States; ^5^Nash Family Department of Neuroscience, Department of Neurology, Friedman Brain Institute, Ronald M. Loeb Center for Alzheimer's Disease, Icahn School of Medicine at Mount Sinai, New York, NY, United States

**Keywords:** cholesterol, APOE, Alzheimer's disease, Parkinson's disease, neurodegeneration, CYP46A1, Huntington's disease (HD)

Within the central nervous system, maintenance of normal cholesterol homeostasis is important for various processes, including brain development, myelination, and neuronal signaling. These functions depend critically on *de novo* synthesis within the brain, as apolipoprotein particles in the systemic circulation are sequestered from the brain by the blood-brain barrier. While maintenance of whole-body cholesterol levels is well-understood, brain cholesterol homeostasis has emerged as an important topic of study given our rapidly evolving understanding of the interactions among neural cells that govern circuits and behavior, as well as the brain's response to aging neurodegeneration. During brain development, cholesterol is critical for neural differentiation and support across brain regions—needs that are met by high levels of cholesterol synthesis. Into adulthood, cholesterol generation shifts from neurons to predominantly astrocytes, and overall synthesis decreases as the brain ages in specific brain regions (Thelen et al., 2006). Age-related changes in cholesterol homeostasis are hypothesized to underlie reduced neural plasticity and function with age. Moreover, changes in cholesterol metabolism may increase risk for certain forms of neurodegenerative disease (Feringa and van der Kant), as exemplified by several cholesterol-related genes, including *APOE, APOJ, ABCA7*, and *SORL1* (Karch and Goate, 2015), that were found to be associated with Alzheimer's disease (AD) risk. These observations have prompted important questions in recent years related to brain cholesterol homeostasis: what are the causal links between cholesterol dysregulation and age-related neurodegenerative diseases? Are there potential peripheral biomarkers of this dysregulation that may be useful for diagnosing neurodegeneration? What methodological advances can push this field forward? In this Research Topic, “Cholesterol and Neurodegenerative diseases: pressing questions and how to address them,” we present ten studies that collectively address these important questions, which we hope will create a framework for further exploration.

The involvement of cholesterol in the pathogenesis of neurodegenerative diseases is summarized in four review papers for this topic. Feringa and van der Kant present a broad overview of cholesterol function within the brain, as well as putative mechanisms by which cholesterol metabolism may modify pathobiology of AD. The review explores how Alzheimer's risk alleles linked to cholesterol homeostasis may regulate pathogenesis and proposes emerging methods that may shed light on these associations (Feringa and van der Kant). Kacher et al. address a topic of altered brain cholesterol homeostasis in Huntington's disease (HD) and how cholesterol dyshomeostasis could be a determinant factor in neuronal degeneration and HD progression. The pathways and major mechanisms by which cholesterol and sterols are regulated in the CNS are presented alongside the main clinical strategies for restoring cholesterol metabolism in the CNS in HD. Pikuleva and Cartier focus on CYP46A1, a key enzyme for cholesterol elimination from the brain, and provide a summary of seminal research that led to the identification of CYP46A1 as a therapeutic target for major neurodegenerative (including AD and HD) and non-neurodegenerative brain disorders. The authors describe CYP46A1 involvement in critical cellular pathways beyond cholesterol homeostasis (e.g., gene transcription, endocytosis, misfolded protein clearance, vesicular transport, and synaptic transmission) and propose how a single enzyme can exert central control of essential brain functions. Duong and colleagues review vascular contributions to cognitive impairment (VCID) and dementia and link cholesterol, atherosclerosis, APOE, and VCID into a model. The authors then discuss potential future therapies for both atherosclerosis and dementia as a result of vascular pathology (Duong et al.). The review portion of the topic is concluded by an intriguing analysis of the research workforce studying neurodegeneration and cholesterol. Pfrieger uses a novel bibliometric TeamTree approach to identify key players in this research area, while demonstrating how the field has developed since the 1950s.

The topic's experimental papers focus on the identification of potential biomarkers for PD as well as cognitive function: four studies used patient serum or plasma and one used cerebrospinal fluid. For example, Bakeberg et al. examined serum HDL, LDL, cholesterol, and triglycerides in subjects from the Australian PD Registry, finding a sex-specific elevation in HDL that associates with worse cognitive function in female PD subjects compared to males. Griffiths et al. conducted sterol profiling in the cerebrospinal fluid of PD patients to identify cholesterol metabolites or pathways linked to PD. This work highlights the potential clinical significance of the bile acid biosynthesis pathway in PD and defines a methodology that can be used to measure the pathway intermediates within a clinical laboratory setting. Simeone et al. investigated the association of cardiovascular risk with cognitive function, reporting that serum levels of PCSK9 are associated with short-term memory only in females with high cardiovascular risk. Liu et al. analyzed whether long-term increase or decrease in plasma cholesterol levels is associated with cognitive decline. The study found that the long-term increase in non-high-density lipoprotein cholesterol was associated with decreased risks of both global cognitive decline and memory decline in females and participants without any cardiovascular disease. Finally, McFarlane et al. assessed plasma or serum lipids as biomarkers of early cognitive decline in aging adults (McFarlane et al.). Significant differences were found only for the serum levels of total cholesterol and low-density lipoprotein cholesterol with both being the highest in the mild cognitive impairment group and lowest in the mild dementia and cognitively normal groups.

Overall, this special topic highlights the key molecular pathways involved in each major aspect of cellular cholesterol metabolism, the connection to pathogenesis of neurodegenerative diseases, and points to new directions for the field. Since 2000, research in this field has expanded considerably, and therapeutic strategies have begun to emerge. However, pressing questions remain to be answered. In particular, how is cholesterol metabolism affected in specific neural cells, and what new tools can provide insights? Generation of various neural cell types from human induced pluripotent stem cells, combined with introduction of specific risk mutations by CRISPR/Cas9 gene-editing will provide a comprehensive new tool with which to study cholesterol metabolism in the setting of disease. Single-nuclei or single-cell RNA-sequencing of post-mortem tissues will discriminate the specific imprinting of neural cells related to cholesterol metabolism in pathological contexts. Finally, study of the biological role of cholesterol derivatives would broaden our knowledge of neurodegeneration and could lead to potential biomarkers. Combined, these future studies will require significant efforts but will likely have a significant impact on targeting cholesterol homeostasis to slow down neurodegenerative processes.

## Author Contributions

All authors conceptualized and wrote the manuscript. All authors approved the submitted version.

## Conflict of Interest

The authors declare that the research was conducted in the absence of any commercial or financial relationships that could be construed as a potential conflict of interest.

## Publisher's Note

All claims expressed in this article are solely those of the authors and do not necessarily represent those of their affiliated organizations, or those of the publisher, the editors and the reviewers. Any product that may be evaluated in this article, or claim that may be made by its manufacturer, is not guaranteed or endorsed by the publisher.
